# Thrombo-Inflammation: A Focus on NTPDase1/CD39

**DOI:** 10.3390/cells10092223

**Published:** 2021-08-27

**Authors:** Silvana Morello, Elisabetta Caiazzo, Roberta Turiello, Carla Cicala

**Affiliations:** 1 Department of Pharmacy, University of Salerno, 84084 Fisciano, Italy; smorello@unisa.it (S.M.); rturiello@unisa.it (R.T.); 2 Department of Pharmacy, School of Medicine and Surgery, University of Naples Federico II, 80131 Naples, Italy; elisabetta.caiazzo@unina.it; 3 PhD Program in Drug Discovery and Development, University of Salerno, 84084 Fisciano, Italy

**Keywords:** CD39, CD73, adenosine, inflammation, platelets, thrombosis, adenosine, ADP, ATP, COVID-19

## Abstract

There is increasing evidence for a link between inflammation and thrombosis. Following tissue injury, vascular endothelium becomes activated, losing its antithrombotic properties whereas inflammatory mediators build up a prothrombotic environment. Platelets are the first elements to be activated following endothelial damage; they participate in physiological haemostasis, but also in inflammatory and thrombotic events occurring in an injured tissue. While physiological haemostasis develops rapidly to prevent excessive blood loss in the endothelium activated by inflammation, hypoxia or by altered blood flow, thrombosis develops slowly. Activated platelets release the content of their granules, including ATP and ADP released from their dense granules. Ectonucleoside triphosphate diphosphohydrolase-1 (NTPDase1)/CD39 dephosphorylates ATP to ADP and to AMP, which in turn, is hydrolysed to adenosine by ecto-5′-nucleotidase (CD73). NTPDase1/CD39 has emerged has an important molecule in the vasculature and on platelet surfaces; it limits thrombotic events and contributes to maintain the antithrombotic properties of endothelium. The aim of the present review is to provide an overview of platelets as cellular elements interfacing haemostasis and inflammation, with a particular focus on the emerging role of NTPDase1/CD39 in controlling both processes.

## 1. Introduction

Experimental and clinical data demonstrate that inflammation may cause haemostatic aberration, leading to thrombosis. On the other hand, thrombosis may expand inflammation; in this way, a vicious cycle is established between the two processes, which increase tissue damage and cardiovascular risk [1,2]. To define the important link between innate immunity and thrombosis, the term of “immune-thrombosis” has been coined: it refers to thrombosis as a physiological process, that is part of host defence and defines an effector mechanism of innate immunity [3].

The crosstalk between inflammation and thrombosis passes through the interaction among several cells including platelets, monocytes, macrophages, talking to each other and all with endothelial cells. The endothelium is the first site where haemostasis and inflammation communicate, through several connecting points. When the endothelium is intact, under physiological conditions, the balance between its pro-and anti-thrombotic features is preserved; on the contrary, when the endothelium is activated, such as during inflammation, it loses these properties and becomes a suitable surface where inflammatory events build up a prothrombotic environment [1,2,4,5]. The knowledge of cellular and molecular events interfacing inflammation and haemostasis is required to identify novel therapeutic strategies. Here, we highlight the emerging role of NTPDase1/CD39 in the crosstalk between inflammation and thrombosis. NTPDase1/CD39 is an ectonucleotidase hydrolysing ATP to ADP and AMP; it is highly expressed on vasculature, platelets, and immune cells and is able to control thrombotic and immune processes within an inflammatory environment [6,7,8].

## 2. Platelet Activation

Platelets play a central role in haemostasis, thrombosis, and atherosclerosis; they are crucial in the primary haemostatic defence mechanism through adhesion to damaged vessels and the following activation. However, beyond their role in haemostasis and thrombosis, platelets were also described long time ago as “inflammatory cells” [9]. Over three decades, there has been growing evidence for platelet contribution in inflammatory disorders and now their involvement in both aspects of thrombosis: innate immune response and haemostatic disorders is widely documented [1,2,3,10].

Normally, platelets circulate freely in the blood, protected from activation by the antithrombotic properties of vascular endothelium, including the release of prostacyclin and nitric oxide, the NTPDase1/CD39 pathway and the expression on its surface of negatively charged molecules, such as glycoproteins, glycosaminoglycans, chondroitin sulphate and heparan sulphate, which contribute to build up on-thrombogenic endothelial surfaces [11]. The stability of platelet inactive state is also preserved by organized phospholipid disposition in their inner plasma membrane. However, a damaged endothelium, following inflammation, hypoxia or altered blood flow, becomes activated, losing its anti-aggregating and anti-coagulant properties and promoting platelet activation [10].

Activated platelets express on their surface molecules driving platelet–endothelium adhesion and platelet–leukocyte interaction, events that are crucial for platelet engagement into the inflammatory environment. Following activation, P-selectin (CD62P), normally stored in α granules, translocates onto platelet surfaces and engages its receptor P-selectin glycoprotein-1 (PSGL-1) on polymorphonuclear leukocytes (PMNs) and monocytes; the interaction of platelets with leukocytes may result in local fibrin deposition through an increased tissue factor (TF) expression in these cells [12,13,14,15] which, in turn, will promote blood coagulation. Platelet-leukocyte interaction is a key event bridging inflammation with thrombosis [16]. Neutrophils are inflammatory cells representing the first defence line during acute inflammation; they are recruited to an inflammatory site by chemotactic factors. In addition, a further neutrophil recruitment in an inflammatory site occurs when platelets are bound to endothelial cells; in this way, neutrophils interact with platelets and then with endothelial cells. Thus, platelets induce neutrophil activation leading to neutrophil extracellular trap (NET) formation. There is evidence that the interaction between platelet-derived P selectin with PSGL-1 on neutrophils is a crucial event for NETosis that, in turn, strongly contributes to immunothrombosis [17].

The platelet receptor GPIbα, engaged on platelet surface following activation, binds to P-selectin and von Willebrand factor (vWF) externalized by endothelial cell granules following vascular damage. Other adhesive proteins that have been described on platelets, and are important for cell–cell interaction, are CD40 and CD40L. On activated platelet surface, CD40L by binding to CD-40 (its counter-receptor on endothelial cells and monocyte/macrophages), promotes platelet-monocyteand platelet-endothelial cell interactions. The interaction between CD40L-CD40 promotes cell–cell adhesion but also regulates several functions in monocytes, such as chemokine and cytokine secretion, expression of TF, upregulation of adhesive receptors and differentiation of monocytes into macrophages [10,18,19,20].

Another important molecule promoting platelet adhesion to the endothelium and monocytes is C reactive protein (CRP), an acute phase protein whose synthesis in the liver is under cytokine control. CRP has also been found in the walls of damaged vessels and in atherosclerotic plaques [21]. CRP consists of five identical subunits of 206 amino acids each; pentameric CRP can be dissociated in monomers either in vitro or in vivo. Pentameric and monomeric forms of CRP possess different biological activity; the pentameric CRP by binding to FcγRIIa (CD32) expressed on platelets [22] inhibits platelet-endothelial cell interactions [23]. On the contrary, monomeric CRP by binding to FcγRIII (CD16) on platelets promotes platelet–neutrophil interaction [22,24]. Furthermore, activated platelets convert pentameric CRP to monomeric. Thus, it is worth noting that CRP regulates platelet activation, and, in turn, platelet activation regulates the conformational status and biological function of CRP. Monomeric CRP (through platelet activation) may lead to monocyte activation, thus representing an important mechanism linking platelet/monocyte activation and invasion of the vascular wall [25,26]. In addition, CRP inhibits the fibrinolytic pathway by reducing the release of tissue plasminogen activator (t-PA) and by stimulating the release of plasminogen activator inhibitor-1 (PAI-1) from endothelial cells [26,27].

Thus, platelets participate in physiological haemostasis, but also in inflammatory and thrombotic events occurring in an injured tissue. However, while physiological haemostasis develops rapidly to prevent excessive blood loss, in an endothelium activated by inflammation, hypoxia of by altered blood flow, thrombosis develops slowly.

The inflammatory environment is enriched in cell-derived platelet agonists, such as arachidonic acid, thromboxane, thrombin, platelet activating factor. Activated platelets release the content of their granules including ATP and ADP released from their dense granules [10]. NTPDase1/CD39 dephosphorylates ATP to ADP and to AMP that, in turn, is hydrolysed to adenosine by CD73.

NTPDase1/CD39 has emerged has an important molecule into the vasculature and on platelet surface, it limits thrombotic events and contributes to maintaining the antithrombotic properties of endothelium [11,28]. The aim of the present review is to provide an overview of platelets as cellular elements interfacing haemostasis and inflammation, with a particular focus on the emerging role of NTPDase1/CD39 in controlling both processes.

## 3. Nucleotides and Signaling

Extracellular nucleotides are released by a wide variety of cells, including activated platelets, endothelial cells, smooth muscle cells, leukocytes; they signal through P2X receptors (P2 X 1 − P2X7), that are ligand-gated ion channel, and P2Yreceptors (P2Y1, P2Y2, P2Y4, P2Y6, P2Y11–P2Y14), that are G-protein coupled receptors. Released ATP is hydrolysed to ADP and AMP by ecto-nucleoside triphosphate diphosphoydrolases (apyrase, NTPDases); AMP, in turn, is hydrolysed to adenosine by ecto-5′-nucleotidase (CD73) [29].

Eight members of NTPDase, all differently localized, have been identified: NTPDase 1, NTPDase 2, NTPDase 3 and NTPDase 8 are cell-surface-located enzymes. NTPDase 4, NTPDase 5, NTPDase 6 and NTPDase 7 are localized intracellularly; however, soluble forms for NTPDase 5 and NTPDase 6, although not enzymatically active, have also been described. A soluble form (enzymatically active) of NTPDase1/CD39 has been found in microparticles in human plasma. AMP derived from ATP and ADP hydrolysis can be in turn converted into adenosine by CD73, an ecto-5′nucleotidase anchored to cell membrane, largely distributed in all tissues and expressed by a wide cell variety. CD73 also exists in a soluble form, enzymatically active. CD73 catalyzes an irreversible reaction, leading to adenosine accumulation into the extracellular environment.

CD39 enzymatic activity determines the rate of ATP/ADP hydrolysis to AMP. The conversion of AMP to adenosine by CD73 enzymatic activity underlies the overall role of CD39 [29,30].

Adenosine derived from AMP hydrolysis, through CD73 activity, signals through P1 receptors. A_1_, A_2A_ and A_2B_ are high affinity receptors, while A_3_ is low affinity receptor. Adenosine A_2A_ receptor is coupled to adenylate cyclase, and it is the receptor mostly involved in the adenosine-mediated antiplatelet and anti-inflammatory effects [31,32].

## 4. NTPDase1/CD39 and Vascular Inflammation

NTPDase1/CD39 is a ubiquitous enzyme present on the surface of a variety of cells, including platelets, endothelium and leukocytes and represents the dominant ectonucleotidase in the vasculature. Among all NTPDase members, NTPDase1/CD39 plays the major role in controlling the immune response, vascular inflammation and thrombosis and there is increasing evidence that its expression on endothelial cells represents a further contribute to endothelium antithrombotic properties, together with nitric oxide, prostacyclin, heparan sulphate molecules [6,11,16,33].

Released nucleotides control several cellular functions modulating cellular homeostatic responses. On platelets, ADP triggers aggregation through P2Y1 and P2Y12 activation; P2Y1 is coupled to a G_q_-protein and activates a phospholipase C while P2Y12 is coupled to a G_i_-protein and inhibits adenylate cyclase [34]. Hence, levels of NTPDase1/CD39 cellular expression control P2Y1 and P2Y12 receptor activation. On these bases, the purinergic machinery, CD39/CD73 adenosine pathway and signalling, being activated as an early event following tissue damage, controls cell to cell communication and tissue homeostasis [35].

**Figure 1:**
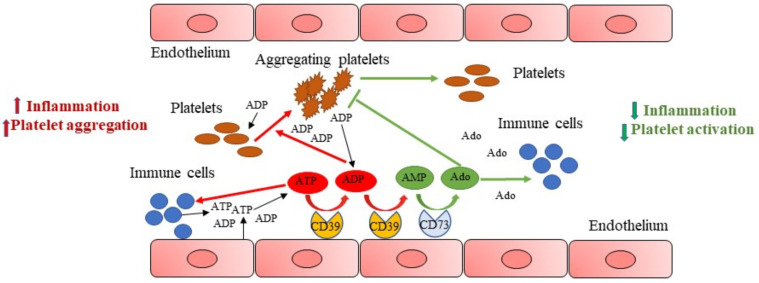
NTPDase1/CD39 and vascular inflammation. After tissue damage, levels of extracellular ATP strongly increase, contributing to boost inflammation, and together with ADP promote platelet activation and aggregation (red lines). Endothelial cell CD39 by hydrolysing ATP to ADP and to AMP, reduces ADP concentrations at the site of injury. CD73 then hydrolyses AMP to adenosine, that inhibits platelet aggregation and reduces inflammation (green lines), providing to maintain tissue homeostasis.

Under physiological conditions, nucleotides are released from vascular cells at low rates; in an inflammatory environment, levels of extracellular ATP strongly increase, after tissue damage and, together with ADP, released from platelet dense granules [10,36], function as autocrine and paracrine signalling molecules and contribute to stimulate other platelets to accumulate at the site of injury and to aggregate (Figure 1).

NTPDase1/CD39 at vascular level, by hydrolysing ATP to ADP and to AMP, reduces ADP concentration at the site of injury (Figure 1) and controls P2 and P1 receptor activation. Hence, CD39 has been considered a key modulator of thrombus formation. The contribute of ATP hydrolysis toward CD39 antithrombotic effect is poor. ATP activates P2X1 receptor on platelets, causing reversible shape change, but not platelet aggregation [37]. ATP, through P2X1 platelet receptor activation, plays a role in platelet adhesion to collagen and can synergize with ADP to activate platelets, especially under shear stress conditions [38]. It has been shown that the rate of ATP hydrolysis by CD39 is not sufficient to prevent local P2X1 activation by micromolar ATP concentrations [39].

Additionally, CD73 hydrolyses AMP to adenosine which, instead, inhibits platelet aggregation and exerts anti-inflammatory effects [35] (Figure 1).

Thus, in an inflammatory environment, ATP promptly released following cellular stress is pro-inflammatory, functioning as “danger sensor” into the tissue; conversely, adenosine is an anti-inflammatory signal, providing to maintain tissue homeostasis. CD39 and CD73 expression and activity regulate levels of extracellular nucleotides and nucleosides; these ectoenzymes work in tandem to transform a pro-thrombotic and proinflammatory environment rich in ATP and ADP, in an anti-thrombotic and anti-inflammatory environment, rich in adenosine [40,41].

In the vasculature, the increase in nucleotide bioavailability results in vascular effects due to P1 and P2 receptor activation, such as apoptosis, fibrosis, vasoconstriction or vasorelaxation, platelet aggregation, inflammation, and vascular permeability [33,42,43,44,45]. The loss of CD39 on activated endothelium sustains platelet aggregation and thrombogenesis, resulting in enhanced platelet P2Y1 and P2Y12 activation [6,46].

Pathological conditions characterized by increased cardiovascular risk might be associated with a reduced CD39 expression and/or activity [47,48,49]. Conversely, increased expression of CD39 on endothelium and inflammatory cells, working in tandem with CD73, might cause inhibition of platelet activation by increasing extracellular adenosine levels and may be associated with a reduced risk of thrombosis [50,51,52]. In a murine model of hypothermia, increased platelet activation and prothrombotic events were seen to be associated to reduced NTPDase1/CD39 enzymatic activity and could be prevented by soluble CD39 administration [53].

We have found that platelets from female rats have increased CD39 expression and activity compared to male platelets and, in concomitance, a reduced reactivity to ADP [54]. Mice genetically deficient in CD39 show a prothrombotic phenotype and develop a large infarct size in the model of ischemia–reperfusion injury [55,56,57].

Hypertension is a main cause of endothelial damage and cardiovascular risk [58]. To evaluate the impact of hypertension on CD39 vascular expression, Roy and co-workers performed experiments in animal models [59]. Interestingly, they found that CD39 mRNA expression was downregulated in resistant arteries and thoracic aorta and inversely correlated with cardiac remodelling, in both angiotensin II (ANGII) and deoxycorticosterone acetate and NaCl 1% (DOCA salt) induced hypertension. Furthermore, they demonstrate that the persistence of hypertension slowly reduces CD39 aortic expression and activity; such a reduction in vascular CD39 expression and functionality might be a cause of platelet activation, increased vascular permeability and inflammatory cell accumulation [59]. CD39-null mice develop pulmonary arterial hypertension and remodelling, and ventricular hypertrophy, following hypoxia [60].

In recent years, much attention has been given to atherosclerosis as a chronic inflammatory disease characterized by vascular infiltration of innate immune cells. On this respect, it is worth noting that P2Y12 receptor is also involved in platelet/monocyte aggregate formation, an event linking thrombosis with vascular inflammation and atherosclerosis [59,61]. Such evidence further strengthens the importance of CD39 in atherogenesis [62,63]. Consistently, drugs inhibiting P2Y12 receptor are beneficial in experimental models of atherosclerosis and reduce platelet/monocyte aggregate formation induced by bacterial lipopolysaccharide in humans [64,65,66].

The role of CD39 in atherosclerosis appears to be complex. While it has been demonstrated that CD39 hemizygous mice (*CD39* +/-) on ApoE knockout (KO) background mice (*ApoE* -/-) develop severe atherosclerotic lesions compared to *CD39* +/+ *ApoE* -/- genotype mice, double knockout mice, *ApoE* -/- and *CD39* -/-, exhibit reduced atherosclerotic lesions compared to *ApoE* -/- mice [67]. This effect might be the result of platelet hyporeactivity due to P2Y1 receptor desensitization following prolonged ADP accumulation [68] but may also be due to a decrease in plasma cholesterol levels and/or an increased cholesterol efflux from macrophages and reduced foam cell development [67,69]. Probably, multiple factors contribute to the atheroprotective phenotype of double knock-out mice.

Experimental data are corroborated by clinical observations. In patients’ stenotic aorta valve has been demonstrated a diminished CD39 activity, that consequently could be responsible for reduced ATP hydrolysis on fibrosa surface of stenotic valve, increasing the thrombotic risk and vascular inflammation, accordingly [70].

CD39 has also been found in platelet microparticles where it contributes to control thrombosis. It has been observed that while in microcirculation prevails nucleotide hydrolysis by endothelial NTPDase1/CD39, in larger vessels nucleotides are equally metabolized by surface and soluble ENTPDase [71,72,73].

Soluble NTPDase1/CD39 is up regulated under exercise conditions in humans; it may represent a homeostatic mechanism counteracting the thrombotic risk due to an increased ADP release during exercise [74].

Increased NTPDase1/CD39 activity in circulating microparticles has been found in patients with idiopathic pulmonary arterial hypertension, while in patients’ lung vasculature there is reduced CD39 expression [60,75]. Authors hypothesise that this may be a mechanism of control of platelet aggregates. Furthermore, there is a correlation among ATP and ADP plasma levels in patients with peripheral artery disease (PAD) and risk factors for PAD [76]. In PAD patients, authors found a positive correlation among systolic blood pressure, triglycerides, and ADP levels, and among cholesterol levels, ATP levels and soluble CD39 activity. The reduced CD39 activity only in patients with critical ischemia suggests that such a reduction contributes to disease progression among PAD patients. Instead, in smoking subjects with PAD, they found increased ATP and ADP levels together with increased CD39 activity compared to smoking subjects without PAD; the authors suppose that in absence of increased CD39 activity, smoking patients would have even higher ATP and ADP levels [76].

## 5. NTPDase1/CD39 Bridging Inflammation and Thrombosis

As discussed above, inflammation and thrombosis are two tightly connected processes whose main players are platelets, innate immune cells, and endothelial cells.

Evidence that NTPDase1/CD39 controls several immune functions but also haemostasis has pointed to a role for this ectoenzyme as a boundary line between inflammation and thrombosis. It is evident that deep knowledge of factors controlling CD39 expression and activity together with the knowledge of cellular functions controlled by CD39 are of fundamental importance to unravel the role of CD39 at the interface between inflammation and thrombosis.

Inflammatory cytokines, tumour necrosis factor (TNF) alpha, interleukin (IL)-1beta, IL-6, tumour growth factor (TGF)beta 1, downregulate vascular CD39 expression with the consequent ADP-induced platelet recruitment and increased platelet/leukocyte interaction [59,62]. Superoxide release following endothelial depolarization inhibits the NTPDase1/CD39 activity [77]. Interestingly, into the milieu of an atherosclerotic plaque the loss of CD39 expression might be synergic with the reduced CD39 functionality due to high oxidative environment. Mechanical stress and flow disturbances are also key factors controlling CD39 expression and activity [78].

We have shown that IL-17A exacerbates ferric chloride-induced arterial thrombosis in rats through the downregulation of CD39 expression in the carotid artery [79]. Interleukin 17A may play a role in the increased cardiovascular risk associated to chronic inflammatory diseases, such as rheumatoid arthritis, psoriasis, inflammatory bowel diseases [80]; its effect may be correlated to a decrease in CD39 activity and expression [79]. There is evidence that the immunoregulatory role of endogenous IL-27 passes through CD39 expression [81]. Recently, it has been found that an antibody neutralizing IL-27 reduces CD39 expression on human monocytes and this may be the main cause of increased frequency of IL17^+^ T cell proliferation following blockade of IL-27 [82].

The CD39 expression levels are also linked to the cardiovascular risk associated with immune and autoimmune diseases. In an experimental model of lupus in mice it has been hypothesised that CD39 may have a protective role in vascular disease associated with lupus, the effect could be dependent upon the suppression of neutrophil extracellular traps release (NETosis) due to adenosine [49]. The antiphospholipid syndrome (APS) is an autoimmune disorder in which antiphospholipid antibodies are implicated in the development of arterial and/or venous thrombosis. APS is present in one third of lupus patients, or it may be primary. A protective role of CD39 associated with a reduction of TF decidual expression has been demonstrated in a murine model of antiphospholipid antibody-induced miscarriage [83]. In agreement, it has been shown that agonism to adenosine A_2A_ inhibits NETosis of APS patients’ neutrophils and in a murine model of APS protects from venous thrombosis [84].

Notably, in humans, genetic variants of E-NTPDase1/CD39 may represent risk factors for diseases such as inflammatory bowel disease (IBD), AIDS and diabetes [47,48,85].

Little is known by researchers about the role of CD39 in venous thrombosis. Into the venous bed, blood flow is lower than into the artery bed, atherosclerotic plaque does not develop, and coagulation factors play a preeminent role in driving thrombosis [86]. In veins, activation of the endothelium by altered blood flow, inflammation, hypoxia, is associated to a reduction of anticoagulant factors and increased expression of thrombogenic molecules, acquiring a procoagulant phenotype; platelets are also involved playing a role in cell recruitment and activation [3,86]. Recently, it has been demonstrated that, in mice, the partial deficiency of CD39 (*CD39* +/- mice) contributes to venous thrombosis under flow restricted conditions [8]. *CD39* +/- mice show increased fibrin deposition within the vessels and increased tissue factor in thrombus lysate [8]. Interestingly, the authors demonstrate that CD39 deficiency increases neutrophil activation and NET formation. Increased circulating platelet/neutrophil hetero-aggregates have been found in CD39-null mice [8,87]. Such findings demonstrate that CD39 is crucial to mitigate the thrombo-inflammatory response under reduced blood flow conditions [8,87]. In humans, it has been demonstrated that an *ENTPD1* polymorphism is associated to increased venous thromboembolism (VTE) risk and with enhanced platelet response to P2Y1 agonists. The relevance of such evidence lies in the finding that a polymorphism links VTE risk with increased platelet aggregation, supporting the debated role of platelets in VTE pathogenesis [88,89].

Experimental sepsis is a model where inflammatory events and hemo-coagulative disorders strongly overlap. The model is widely used to investigate the link between inflammation and thrombosis [90,91]. Disseminated intravascular coagulation (DIC) and multi organ failure (MOF) are the extreme consequence of sepsis [92]. In an experimental model of murine sepsis, Csoka and co-workers demonstrated that mice treatment with the CD39 mimic apyrase reduces mice mortality and systemic inflammation and organ injury; the beneficial effect of apyrase administration was confirmed by evidence that CD39 genetically deficient mice underwent to severe sepsis symptoms more than control mice [7]. On the other hand, during sepsis, CD39 tissue expression is upregulated. Such evidence strongly suggests that CD39 is an inducible protective pathway in inflammation.

## 6. Targeting NTPDase1/CD39 in Thrombosis

**Figure 2:**
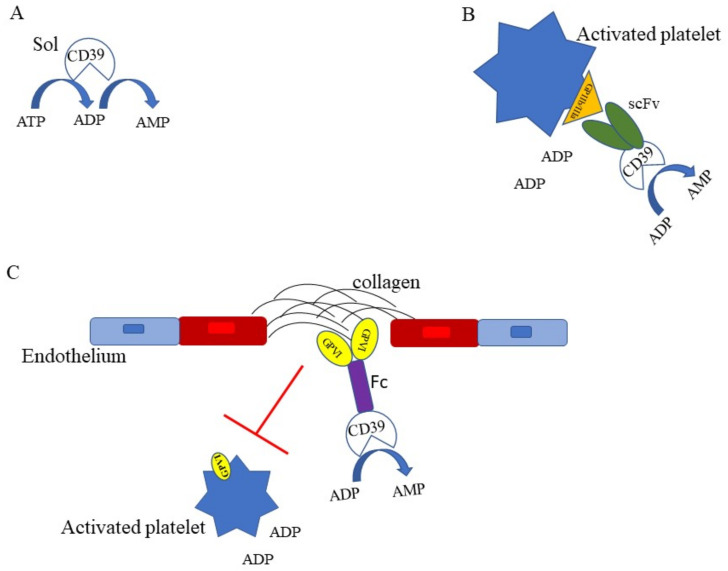
Targeting NTPDase1/CD39 in thrombosis. (**A**) Recombinant soluble CD39 (solCD39) [39,53,94] through its ATPase and ADPase activities inhibits ADP-mediated platelet activation and aggregation. (**B**) CD39, fused with a single-chain antibody (scFv) specific for GPIIb/IIIa [94,95,96], expressed on activated platelets, contributes to reduce ADP concentration, providing strong anti-thrombotic effects. (**C**) The glycoprotein VI (GPVI)Fc-fusion protein is combined to soluble CD39 [97]. The GPVI-Fc inhibits the interaction of GPVI expressed on platelets with vascular collagen in plaques while CD39 reduces the concentrations of ATP/ADP, resulting in a strong inhibition of platelets adhesion and aggregation.

Several attempts have been done to target CD39 as a therapeutic anti-inflammatory and antithrombotic agent. Indeed, under some circumstances targeting CD39 may be crucial to interrupt a self-amplifying mechanism that starting from tissue damage/inflammation activates haemostasis/thrombosis that, in turn, expands inflammation. One approach has been to employ soluble CD39 (solCD39) as an anti-thrombotic agent [39,53] (Figure 2A). The unwanted effect of a therapy with CD39 is the bleeding complication. Strategies have been developed to avoid bleeding, such as recombinant solCD39 fused with crucial residue of PSGL-1, the receptor for P selectin on leukocyte surface [93]. Furthermore, to minimize bleeding complications, a single-chain antibody (scFv) specific for activated GPIIb/IIIa, the platelet fibrinogen receptor, linked to CD39 has been developed [94,95] (Figure 2B). Such a strategy could be beneficial in all those inflammatory conditions where platelet activation occurs and contributes to the spreading of the inflammatory lesion. Granja and co-workers (2019) have demonstrated that Targ-CD39, consisting of CD39 fused with a single-chain antibody directed towards activated platelet integrin glycoprotein IIb/IIIa, inhibits sepsis-associated inflammatory events in mice, through the inhibition of platelets, leukocytes, and endothelial cells interaction [96].

A similar approach has been done by fusing CD39 to a glycoprotein VI (GPVI) Fc fusion protein (Figure 2C) that inhibits platelet adhesion to collagen on atherosclerotic plaques while reduces the concentrations of ADP [97]. Following this path, one can imagine that the fusion of CD39 with protein targeting inflammatory molecules, highly expressed in inflamed tissues, might provide high local CD39 concentration/activity that may exert its focused anti-inflammatory/antithrombotic effect.

## 7. Further Perspectives

Considering the critical role of NTPDase1/CD39 interfacing inflammation and thrombosis, an interesting priority for future studies is to unravel the role of NTPDase1/CD39 in the pathophysiology of COVID-19. Severe COVID-19 syndrome due to SARS Cov-2 infection presents coagulation abnormalities similar to sepsis, characterized by disseminated intravascular coagulation (DIC), acute respiratory distress syndrome (ARDS) and MOF [98], frequent in patients with comorbidities such as arterial hypertension or diabetes, and advanced aged [99]. The pathophysiology of coagulation disturbances has not yet been defined, however, it has been observed that SARS Cov-2 infection is characterized by endothelium dysfunction that, as discussed above, is known to be the cause of haemostasis disturbances, increased vascular permeability, ischemia. The sequence of events in COVID 19 patients leads to macrophage and neutrophils infiltrations, NETosis and the “cytokine storm”, characterized by high circulating levels of soluble IL-2 receptor and pro- inflammatory cytokine, IL-1 beta, IL-6, and TNF alpha [100]. It is likely that under these conditions, high levels of ATP are released. A contribute of NETs to thrombotic disorders in COVID 19 patients has been described, since their ability to activate factor XII and initiate coagulative disorders and platelet activation; the P2Y12 receptor may play a role in thrombotic disorders and monocyte activation [101]. In this contest, the role of CD39 in COVID-19 needs to be explored since it is conceivable that CD39 might represent a useful predictive marker and target for therapeutic interventions especially in patients with cardiovascular risk factors.

## 8. Conclusions

Anti-thrombotic strategies prevent thrombosis but may also affect physiological haemostasis, causing bleeding risks. A good therapeutic strategy to prevent thrombo-inflammation is to target immune cell activation and platelets, which are primarily involved in thrombosis.

Further investigation is required to better define the role of NTPDase1/CD39 in thrombo-inflammation and its potential as a prognostic biomarker and/or therapeutic target. However, to date, it is widely documented that this ectoenzyme, together with CD73, represent an important control point of the inflammatory process, contributing to maintain endothelial anti-thrombotic properties.

In conclusion, NTPDase1/CD39 is a key molecule, whose important role cannot be disregarded when thrombo-inflammation is investigated.

## Data Availability

Not applicable.
